# Core Decompression with Local Administration of Zoledronate and Enriched Bone Marrow Mononuclear Cells for Treatment of Non‐Traumatic Osteonecrosis of Femoral Head

**DOI:** 10.1111/os.13100

**Published:** 2021-10-18

**Authors:** Hai‐yang Ma, Ning Ma, Yu‐feng Liu, Yi‐qun Wan, Gui‐qi Liu, Guang‐bo Liu, Hao‐ye Meng, Huo Li, Xin Wang, Chun‐bao Li, Jiang Peng

**Affiliations:** ^1^ Institute of Orthopaedics/Beijing Key Laboratory of Regenerative Medicine in Orthopedics/Key Laboratory of Musculoskeletal Trauma & War Injuries PLA Chinese PLA General Hospital Beijing China

**Keywords:** Bone marrow mononuclear cells, Core decompression, Hip preservation, Osteonecrosis of femoral head, Zoledronate

## Abstract

**Objective:**

To investigate the efficacy and safety of core decompression (CD) with local administration of zoledronate and enriched bone marrow mononuclear cells (BMMCS) for the treatment of non‐traumatic osteonecrosis of femoral head (ONFH).

**Methods:**

A total of 17 patients (30 hips) diagnosed with stage II and III ONFH according to the 2019 revised Association for Research on Osseous Circulation (ARCO) staging criteria from 2012 to 2014 were retrospectively reviewed. The patients received the following therapy: the BMMCs and zoledronate were injected into the necrotic zone, respectively, along with CD. The mean age of the patients was 36.8 years; 14 were men and three were women. All patients included had non‐traumatic ONFH and a minimum follow‐up of 5 years, which ended when total hip arthroplasty (THA) was performed. Imaging modalities, including plain radiography, computed tomography (CT), and magnetic resonance imaging (MRI) were taken pre‐ and postoperatively. Harris hip score (HHS) was used to evaluate the functional outcomes of femoral head necrosis. Kaplan–Meier analysis was adopted to determine the probability of survivorship with THA as the end point in this series of patients. The correlation between radiological progression or THA and related risk factors were further analyzed. All complications were recorded.

**Results:**

With THA as the follow‐up endpoint, All patients were followed up for an average of 69.1 ± 20.5 months (range, 18–95 months). Preoperative imaging found six hips (20%) at ARCO stage II, 14 hips (46.7%) at stage IIIA, 10 hips (33.3%) at stage IIIB. Fourteen hips (46.7%) shown progression radiologically, while six hips (20%) underwent TKA among these patients with hip preservation. The cumulative survival was 80% (95% *CI*, 0.608–905) at 5 years with THA as the end point. HHS improved from 63.3 ± 8.7 preoperatively to 74.6 ± 20.6 postoperatively (*P* = 0.000). Radiological progression was found to be associated with ARCO stage, Japanese Investigation Committee (JIC) type, and corticosteroid exposure (*P* = 0.047; *P* = 0.012; *P* = 0.031). However, no correlation was found between conversion to THA and the known risk factors. No major complication was reported, with only four patients complaining about general weakness and muscle soreness, and all disappeared within 2–3 days.

**Conclusions:**

The novel treatment modality could relieve pain, delay the progression of collapse, which might be an effective and safe method for hip preservation of early and mid‐term ONFH. However, the effect of this method may be related to ARCO stage, JIC type, and corticosteroid exposure.

## Graphical abstract

The diagram of the procedure step by step. A: Extraction of BMMCs; B: Extraction of the vein blood; C: Stratified mixed sample after centrifugation; D: Administration of the zoledronate and enriched BMMCs. Yellow syringe: zoledronate. Red syringe: enriched BMMCs.

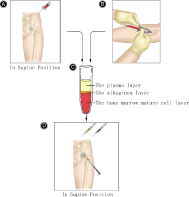

## Introduction

Osteonecrosis of femoral head (ONFH) has become a challenging and devastating disease that mainly affects young peoples^1^, ^2^. ONFH is due to the interruption or damage of the femoral head blood supply, which results in the death of bone marrow and osteocytes, and the following repair procedures lead to structural changes and femoral head collapse^3^. More than 70% of femoral heads with osteonecrosis collapse require total hip arthroplasty (THA) within 2–3 years of diagnosis if not treated^4^. The epidemiological investigation shows that the Asian population is more susceptible to ONFH^5^. Forty per cent of cases undergoing total hip arthroplasty are attributed to ONFH in the Asian population, compared with about 10% in the United States^5^. Even though THAs have shown good performance in young and active populations, implants longevity and the following implant revisions are still outstanding problems among ONFH patients. Thus, hip preservation is necessary, and a variety of joint‐preservation procedures have been introduced^6^.

Core decompression is generally considered to be effective in preventing collapse of the femoral head in the early stage of ONFH^7^, ^8^. Excessive osteoclast activity could weaken the mechanical strength of the repaired region and play an important role in the pathogenesis of femoral head deformity^9^. Therefore, inhibiting the overactivity of osteoclasts may delay the collapse of the femoral head. Bisphosphonate, as a highly potent osteoclast inhibitor, could inhibit the absorptive action of mature osteoclasts during the repair of the infarcted femoral head and have been traditionally used for treating ONFH^10^. Our previous animal studies indicated that bisphosphonate could alter the repair process of ONFH, decrease the femoral head deformity and delay the collapse of femoral head in animal models of ONFH by inhibiting the activity of osteoclasts while suppressing new bone and vessel formation^11^, ^12^. Clinical studies have also shown that core decompression combined with systemic alendronate administration could relieve pain, prevent the collapse of femoral head, or at least defer the need for THA, even in Ficat IIA and III hips^10^, ^13^. However, several reports have documented serious complications including osteonecrosis of the jaw and atypical femur fracture as a result of systemic effects^14^. Aya *et al*. demonstrate that local administration of bisphosphonate could act more accurately on the lesion site, bypass the need for restoring blood flow to the necrotic femoral head and therefore may be preferable to oral or systemic administration^15^. In addition to that, local administration of zoledronate could result in preservation of femoral head structure and prevent femoral head deformity in animal ONFH models as well as avoid the complications caused by systemic administration^3^, ^16^, ^17^, ^18^.

Hernigou *et al*. found that osteogenesis was reduced in corticosteroid‐induced ONFH as a result of decreased mesenchymal stem cells^19^. In a randomized controlled study, Tabatabaee *et al*. found that the clinical symptoms and imaging findings of the bone marrow stem cell injection group were significantly improved compared with the simple core decompression group^20^. A systematic review including 48 studies also found that patients receiving core decompression and regenerative techniques could provide a good clinical effect for the treatment of ONFH and an important improvement in terms of survivorship compared with CD alone^21^. Thus, to supplement stem cells or other osteogenesis factors was necessary for the treatment of ONFH. A sufficient number of cells or osteogenesis factors for transplantation were essential for osteogenesis. Recently, it was found that pretreatment with zoledronate could obviously enhance the proliferation and osteogenesis of BMMCs *in vitro* and *in vivo* ^22^.

Thus, we hypothesized that a combination of local administration of zoledronate and enriched BMMCs could reduce complications, prevent or delay femoral head collapse and THA in patients with ONFH. The aim of this study was to: (i) evaluate the efficiency and safety of the above treatments; (ii) explore the related risk factors of this treatment, and (iii) explore the proliferation and osteogenesis of BMMCs *in vivo*.

## Materials and Methods

All procedures were approved by the Institutional Ethics Review Committee of PLA General Hospital. Informed consent was obtained preoperatively from all patients who participated in this study.

### Inclusion and Exclusion Criteria

Inclusion criteria were as follows: (i) patients with ARCO Stage II, III A, and III B of ONFH; (ii) local administration of zoledronate with enriched BMMCs for treatment of ONFH; (iii) knowledge of preoperative hip function and postoperative hip function; (iv) outcome measures were Harris hip scores (HHS), the ratio of conversion to THA, the ratio of radiological progression, and complications at a minimum follow‐up of 5 years; (v) retrospective study. Exclusion criteria were as follows: (i) patients with traumatic ONFH; (ii) patients with ARCO stage IV; (iii) patients with dysplastic hips, immune system disorders, metabolic disease, or psychiatric disorders.

### Patients

According to the inclusion and exclusion criteria, a total of 17 patients (30 hips) underwent core decompression with local administration of zoledronate and enriched BMMCs from 2012 to 2014 and were retrospectively reviewed. All patients' profiles were assessed by the operating surgeon (first correspondent author) and received a standard preoperative workup, including standard anteroposterior radiographs, computed tomography (CT) scan, and magnetic resonance imaging (MRI) of the affected hip. Each hip's ONFH was staged according to the 2019 revised Association Research Circulation Osseous (ARCO) staging system and classified according to Japanese Investigation Committee (JIC) classification system^23^ based on imaging performance and clinical presentation (Supplementary Tables S1 and S2).

### Surgery

#### Collection and Enrichment of Autologous BMMCs

All patients were operated on by the same senior surgeon (the first correspondent author). After epidural anesthesia and standard prep and draping, bone marrow samples were extracted with a medulla‐puncture needle (6–8 cm in length and about 1.5 mm in diameter) and a 20 mL syringe. The puncture needle was inserted into the marrow cavity of the iliac bone. A quantity of 50–60 mL of bone marrow blood was extracted within one puncture from different directions and depths in a fan‐like shape and infused into the sterile blood storage bag containing sodium citrate and glucose. Other entry points were made within 4–5 cm from the former one until 100–180 mL bone marrow blood was collected. The extracted bone marrow blood was mixed with 200 mL autologous peripheral whole blood and then went through cell sorting under aseptic condition on a COBE 2991TM Cell Processor (GAMBRO BCT. Inc., Lakewood, CO, USA) at 3000 r/min, for 5–10 min. The mixed sample were stratified into the plasma layer, the albuginea layer (containing bone marrow mononuclear cells, white blood cells, etc.), and the bone marrow mature cell layer according to the density gradient, while the BMMCs gradually enrich in the layer of bone marrow mononuclear cells. Different fractions of the mixed sample were output one by one and recovered. The blood volume (VE) (mL) of bone marrow blood containing bone marrow mononuclear cell was determined by two parameters: SV (super out volume, mL/min) and RT (super out rate, min). VE = SV × RT. Therefore, the bone marrow mononuclear cell layer was recovered through manual real‐time control. Bone marrow mononuclear cells were separated and concentrated into 30–50 mL of bone marrow blood for replantation during surgery, and about 2 mL of the transplanted blood sample was retained for bacteriological test. A quantity of 2–5 mL of bone marrow blood samples were taken from every patient before and after separation respectively, 1–2 mL of which went through blood cell count and the number of bone marrow mononuclear cells was counted before and after separation. According to the changes in the volume of bone marrow blood and the number of bone marrow mononuclear cells before and after enrichment, the enrichment efficiency of bone marrow mononuclear cell separation technology was evaluated. Through separation technology, bone marrow mononuclear cells in bone marrow blood were concentrated from (12.2 ± 3.0) × 10^9^/L to (35.2 ± 12.0) × 10^9^/L.

#### Core Decompression and Injection of Enriched BMMCs and Zoledronate

**Fig. 1:**
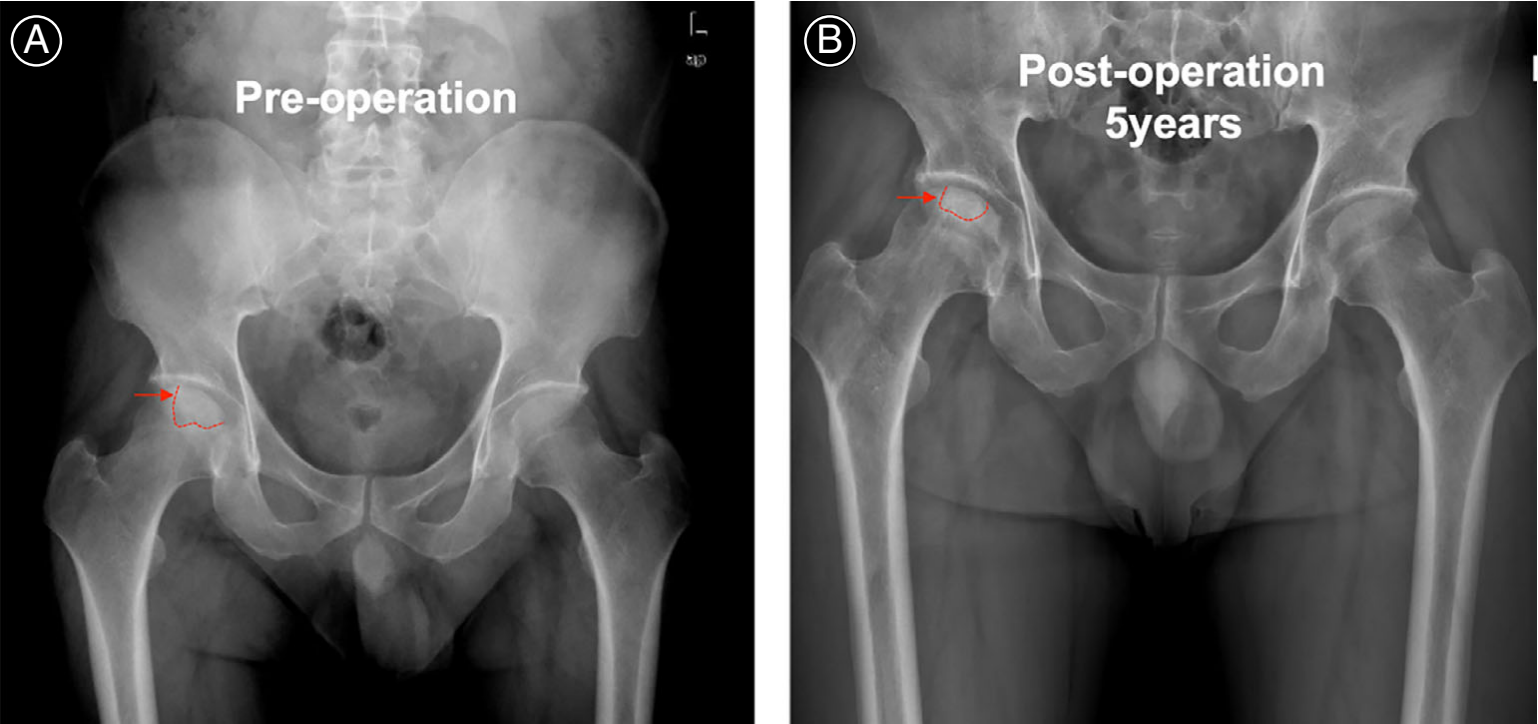
Pelvis X‐ray of osteonecrosis of the right hip shows preserved joint space without imaging progression. The dotted line indicates the necrotic area. (A) AP X‐ray of the hip pre‐operation. (B) AP X‐ray of the hip 5‐year post‐operation.

**Fig. 2:**
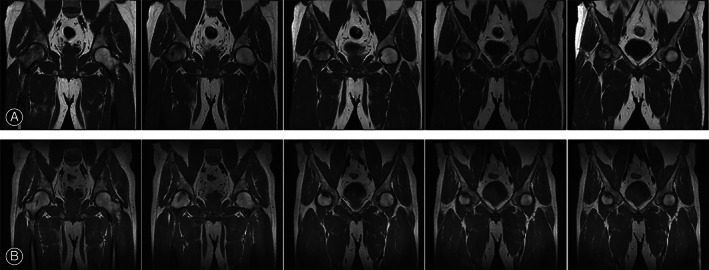
MRI of osteonecrosis of the right hip demonstrates that bone marrow edema obviously disappeared; femoral head remained spherical and no progress in necrotic area of femoral head 5 year later. (A) MRI of osteonecrosis of the right hip pre‐operation. (B) MRI of osteonecrosis of the right hip 5 years post‐operation.

**Fig. 3:**
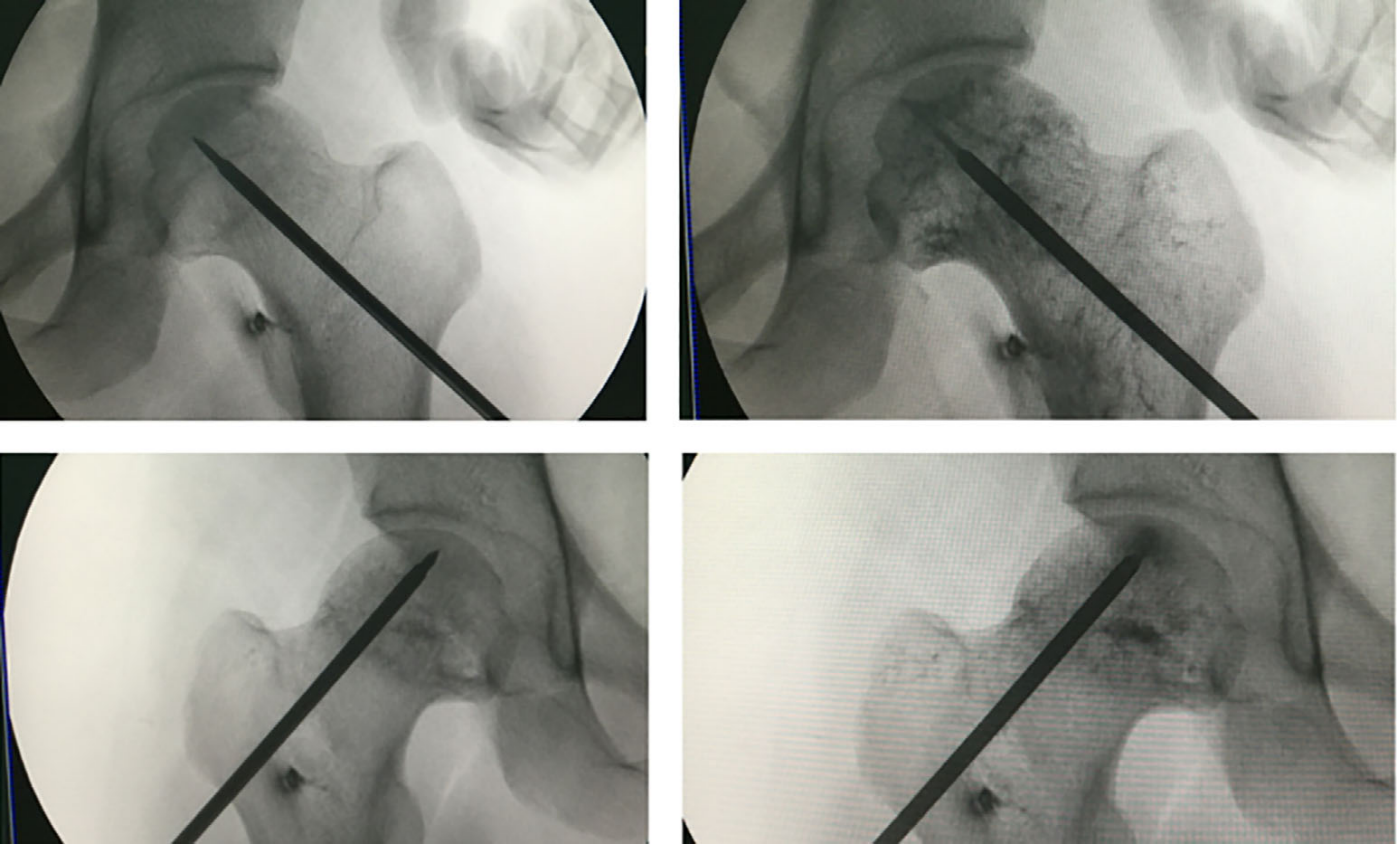
The meglumine diatrizoate was injected into the femoral head under C‐arm image intensifier guidance. It demonstrated that the contrast agent could be distributed into the necrotic area and the venous vessels of femoral head were also displayed.

Patients lied on the orthopaedic traction bed in supine position. At the beginning of core decompression procedure, we carefully identified and evaluated the location, size, and boundary of the necrotic area on AP and lateral radiographs as well as MRI (Figs 1, 2). A 1.0 mm Kirschner wire was drilled into the necrotic lesion of the femoral head 2–3 cm distal to the subchondral bone plate under the surveillance of C‐arm. A 3.5 mm (inner diameter of 1.5 mm) trephine was inserted for core decompression along with the above channel. Two to three holes were bored in total. Then, enriched BMMCs and 200 μg zoledronate were injected into the necrotic zone, followed by injection of 5–10 mL saline. Local administration of meglumine diatrizoate demonstrated that the drug was delivered to the necrotic area of the femoral head under C‐arm image intensifier surveillance (Fig. 3). Finally, the holes for core decompression were sealed with bone wax to prevent leakage. Postoperatively, all patients received prophylactic antibiotic therapy of cefazolin for 3 days.

### Postoperative Rehabilitation

Patients were instructed to walk with crutches and carry protective weight for at least 1 year after operation. Patients were restricted to limited physical activity at the beginning, mainly upper limb strength training with instructions to return to activities gradually. Weight bearing was determined by the surgeon based on the patient's symptom and imaging presentation.

### Outcome Assessment and Follow‐Up

**Fig. 4:**
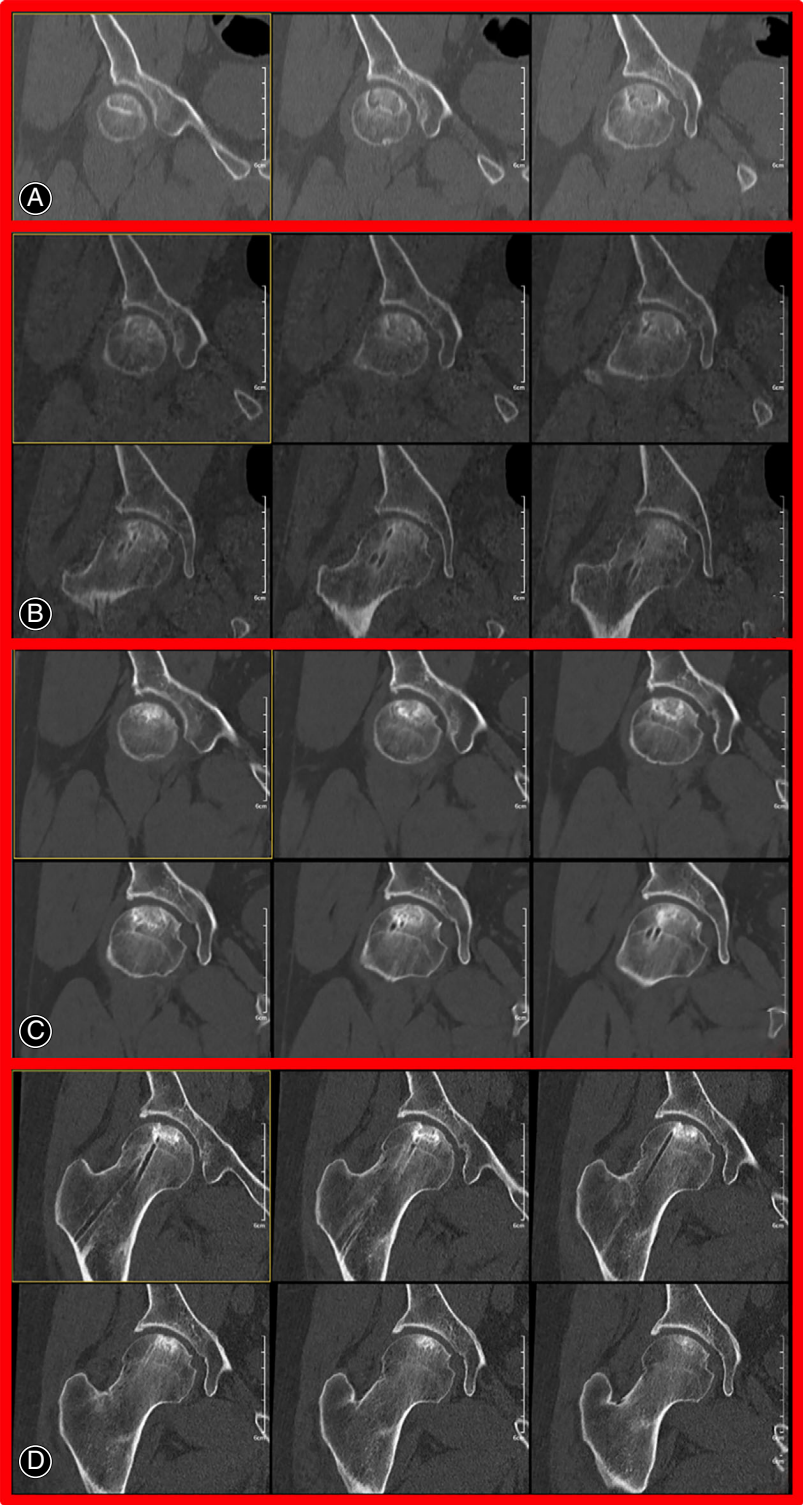
CT of osteonecrosis of right femoral head pre‐ and post‐operation. (A) Obvious necrotic area and bone resorption could be seen pre‐operation. (B) The sclerotic zone gradually disappeared at 16 months. (C) No collapse of the femoral head occurred and obvious hardening in the necrotic area was seen. (D) The outline of the femoral head remained intact at 7 years.

All patients were required to be followed up postoperatively at 3, 6, 12, 18, and 24 months, and every 1 year thereafter. Visiting the outpatient clinic was recommended while the patient's condition was changed. If THA were performed, we would end our follow‐up. Postoperative anteroposterior radiograph of the pelvis, MRI, and CT three‐dimensional reconstruction of the affected hip joints (Fig. 4) were taken to evaluate the condition of the femoral head. Data points were extracted by querying the electronic medical records of patients. The primary outcomes were HHS and conversion ratio to THA. Secondary outcome was radiological progression. All complications were recorded, including both major complications, such as infection, neoplasm development, drug‐related osteonecrosis of the jaw, femur fracture; and minor complications, including superficial wound infection, heterotopic ossification, general weakness, and muscle aches.

### The Indicators

#### Radiological Progression

Radiographs, CT, and MRI were performed to determine ARCO stages of ONFH at the time of each assessment. Radiological progression was decided according to the development of ARCO stage^16^. ARCO stage was an international staging, which was based on imaging performance and clinical presentation. The progression of ARCO stage I, II, or III a to ARCO stage III b or IV on radiological appearance was defined as radiological progression. For stage III b, if the femoral head depression was deeper than before, we also defined it as radiological progression.

#### Harris Hip Score

HHS system has been widely used and is a standard measurement for the hip function. HHS was measured preoperatively and at the last follow‐up (if the patient underwent THA, the HHS before THA was adopted). The system^24^ mainly includes the four aspects of pain, function, absence of deformity, and range of motion. The score standard has a maximum of 100 points. Scores of (90 to 100) are defined as excellent, (80 to 89) good, (70 to 79) fair, or (<70) poor.

#### Survival Analysis

Conversion ratio to THA was designed as the follow‐up end point to evaluate the efficacy of hip preservation. Based on the follow‐up end point, the survival curve was drawn and cumulative survival in 5 years was calculated. Additionally, the time interval from the hip preservation surgery to THA was calculated. We also examined the association of conversion to THA with JIC, ARCO staging, ONFH risk factors, or bilateral hip involvement as well as and radiological progression.

### Statistical Analysis

Statistical analyses were performed using IBM SPSS (version 21.0). Categorical statistics were represented as a number and percentage, while continuous statistics were represented as a mean average and standard deviation. Statistical testing was conducted using Fisher's Exact Test for correlation of categorical variables. Kaplan–Meier analysis was adopted to determine the probability of survivorship in these patients with hip preservation. A *P* value < 0.05 indicated the difference is statistically significant.

## Results

### Demographic and Clinical Characteristics

A total cohort of 30 hips (17 patients) were identified, of which three patients (17.6%) were female and 14 patients (82.4%) were male. All 17 patients were included based on the aforementioned inclusion and exclusion criteria. The mean age of the cohort was 36.8 ± 7.9 years (range, 26–51) (Table 1). Patients' medical history was evaluated for established risk factors for the development to ONFH. Seven hips (23.3%) had inhaled corticosteroid exposure, 16 hips (53.3%) had a history of tobacco exposure, while 12 hips (40%) had a history of excessive alcohol drinking (Table 2). The ARCO classification found on preoperative imaging was Stage II in six (20%) hips, III A in 14 (46.7%) hips, and III B in 10 hips (33.3%). Type B was seen in 3 (10%) hips, C1 in 3 (10%) hips, and C2 in 24 (80%) hips (Table 2) .

**TABLE 1:** Demographic characteristics

Demographics	Value
Age, mean ± SD (range)	36.8 ± 7.90 (26–51)
Gender, *n* (%)	
Male	14 (82.4)
Female	3 (17.6)
BMI, mean ± SD (range)	24.95 ± 3.34 (20.14–31.28)
Patients Treated Bilaterally, *n* (%)	13 (76.5%)
Laterality, *n*	
Left	13
Right	17
Follow‐up duration, mean ± SD (range)	69.1 ± 20.5 (18–95)
Internal time for THA, mean ± SD (range)	36.7 ± 17.1 (18–56)

BMI, Body mass index; SD, standard deviation; THA, Total hip arthroplasty.

**TABLE 2:** Radiological progression and conversion to THA

Pre‐operative risk factors	Radiological progression	NO progression	*P* value	Conversion to THA	NO THA	*P* value
ARCO Stage			0.047			0.196
Stage II	2/6	4/6		0/	6/6	
Stage IIIA	4/14	10/14		3/14	11/14	
Stage IIIB	8/10	2/10		3/10	7/10	
JIC type			0.012			0.220
B	0/3	3/3		0/3	3/3	
C1	0/3	3/3		0/3	3/3	
C2	14/24	10/24		6/24	18/24	
RISK Factors						
Corticosteroid	6/7	1/7	0.031	2/7	5/7	1.000
Smoking	6/16	10/16	0.282	2/16	14/16	0.378
Alcohol drinking	5/12	7/12	0.654	0/12	12/12	0.057
Bilateral hip involvement	13/26	13/26	0.602	6/26	20/26	0.557

THA, Total hip arthroplasty.

### Radiographic Results

As radiological progression at the end point, Stage II—66.7%, Stage IIIA—71.4%, Stage IIIB—20% had no radiological progression; Type B—100%, Type C1—100%, and Type C2—53.3% had no radiological progression.

### Clinical Outcomes

**Fig. 5:**
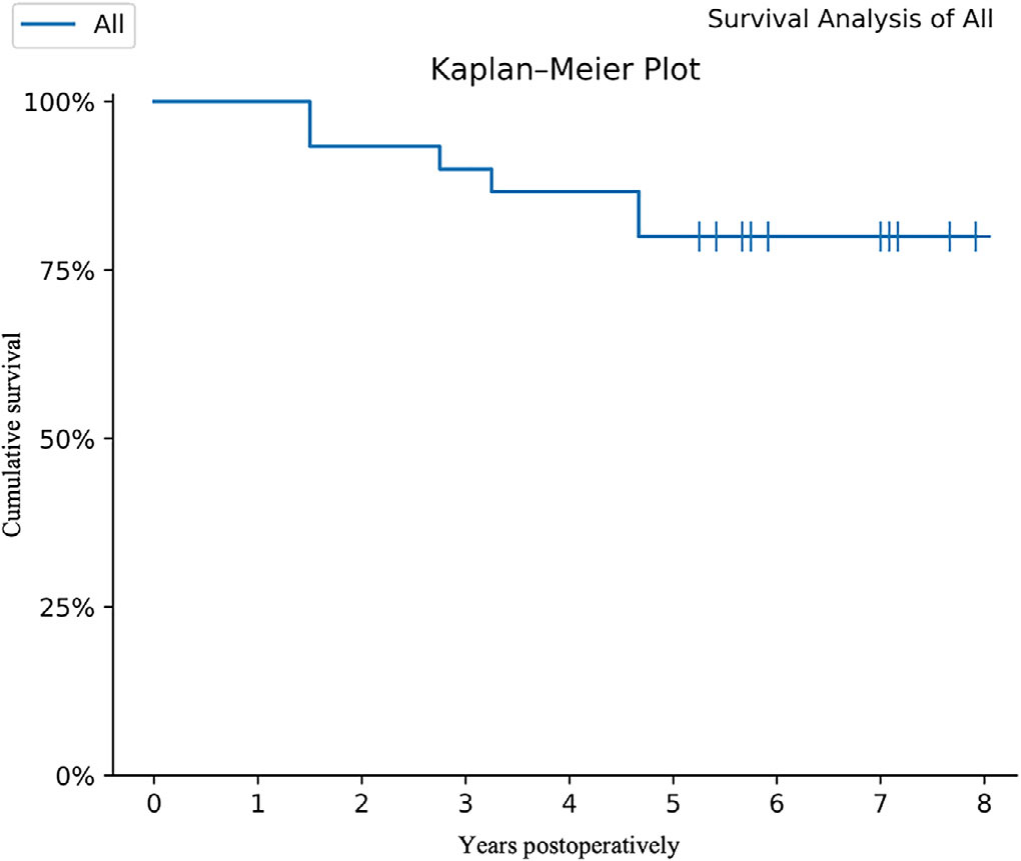
Kaplan–Meier survival curve for hip preservation with THA as the end point.

The mean patient follow‐up was 69.1 ± 20.5 months (range, 18–95 months). The HHS improved from 63.3 ± 8.7 preoperatively to 74.6 ± 20.6 postoperatively (*P* = 0.001). With THA as the end point, 24 hips (80%) had not converted to THA at final follow‐up. Upon further stratification, Stage II—100%, Stage IIIA—78.6%, Stage III B—70% had not converted to THA; Type B—100%, Type C1—100%, and Type C2—75% had not converted to THA (Table 2). With THA as the end point, hip preservation survivorship using the Kaplan–Meier method was 80% (95% *CI*, 0.608%–0.905%) at 5 years (Fig. 5).

We analyzed the correlation between radiological progression and other variables. The correlation between conversion to THA and other variables was also evaluated. Radiological progression was found to be associated with hip ARCO staging and JIC type (*P* = 0.047; *P* = 0.012). The correlation of radiological progression with corticosteroid exposure, was also statistically significant (*P* = 0.031). However, there is no evidence of significant difference between conversion to THA and corticosteroid exposure (*P* = 1.000) (Table 2).

### Complications

There were no major complications, and four patients complained about general weakness and muscle aches within 2–3 days post‐operation, but these symptoms soon disappeared.

## Discussion

To the best of our knowledge, this is the first study to investigate the result of local administration of zoledronate with CD and enriched BMMCs in the treatment of osteonecrosis of femoral head. This study demonstrated relatively good survivability of the novel procedure in ONFH with no major complications at mid‐term follow‐up.

### Efficacy and Safety

In our treatment group, only 46.7% of femoral heads with osteonecrosis present radiological progression, and 20% of femoral heads require prosthetic replacement. However, the literature reported that 70% of ONFH collapse and THAs were required to perform within 3–4 years of diagnosis^4^. When compared with the reported natural course, local administration of zoledronate with core decompression and enriched BMMCs is effective. The JIC classification is considered reliable and effective to predict the collapse of ONFH^25^, ^26^. Studies reported a rate of femoral head collapse of 40% for type B, 80% for type C1, and exceeding 90% for type C2 without intervention^25^, ^27^. The collapse rate was about 60.5% in type C2 hips with other complex preservation surgery^27^, ^28^, ^29^, ^30^, ^31^ (Table 3). In the treatment group, the collapse rate was 58.3%. The collapse rate in our study is similar with these studies^26^, ^27^, ^28^, ^29^, ^30^, ^31^. However, higher technical feasibility, shorter operative duration, and less blood loss are considered to be the advantages of the reported approach. This novel therapeutic approach of CD with local administration of Zoledronate and BMMCS may be beneficial to keep the femoral head spherical structure, or even reverse the disease proceeding^32^. In this retrospective study, there were no related major or minor complications at mid‐term follow‐up. Additionally, ARCO staging and JIC type and corticosteroid exposure were found to be relevant factors affecting hip preservation.

**TABLE 3:** Failure (clinical or radiographic) based on JIC classification system^27^

Study/year	Technique	Hips	Type							
			A		B		C1		C2	
			Hips	Failure	Hips	Failure	Hips	Failure	Hips	Failure
Seyler *et al*. (2008)^31^	CD/NVG/OP1	39	8	1 (12.5%)	12	5 (41.7%)	12	2 (16.7%)	7	5 (71.4%)
Atsumi *et al*. (2006)^30^	PRO	35	11	0 (0%)	20	0 (0%)	4	2 (50%)		
Onodera *et al*. (2005)^29^	TTRO	37					19	9 (47.4%)	18	6 (33.3%)
Nagoya *et al*. (2004)^28^	VIG	35					17	4 (23.5%)	18	15 (83.3%)
Total		146	19	1 (5.3%)	32	5 (15.6%)	52	17 (32.7%)	43	17 (60.4%)

CD, core decompression; NVG, nonvascularized graft; OP1:BMP‐7, Bone Morphogenetic Protein; PRO, posterior rotational osteotomy; TTRO, transtrochanteric rotational osteotomy; VIG, vascularized iliac graft.

### Current Status of Bisphosphonate in ONFH Treatment

Alendronate could inhibit the absorptive action of mature osteoclasts and have been traditionally used for treating ONFH^10^. Kang *et al*. reported that CD combined with systemic alendronate administration could delay THA in a minimum 4‐year follow‐up^13^. A randomized clinical study by Lai *et al*. indicated that alendronate could prevent early collapse in ONFH^10^. However, several reports documented serious complications as a result of systemic effects of this oral drug, such as atypical femur fractures and osteonecrosis of the jaw^33^, ^34^. Local application of zoledronate could inhibit osteoclast activity, block the pathological progression of ONFH, and enhance the mechanical properties of the bone to prevent femoral head collapse^16^, ^17^, ^35^, ^36^. Furthermore, bisphosphonate could reduce marrow edema and remodeling rate^37^, ^38^, ^39^. Previous animal studies have shown that local administration of zoledronate could keep femoral head structure in animal models. Local administration of drugs could achieve the drug distribution of the entire femoral head, and it may be preferable to oral or systemic administration for accurate targeting at the lesion site^15^. In addition, local release of zoledronate could promote bone formation and help avoid complications caused by systemic administration^40^.

### Local Administration of Bisphosphonate and Cell Therapy in ONFH Treatment

Studies have demonstrated that core decompression combined with autologous bone marrow transplantation is effective for treating early‐stage non‐traumatic ONFH^41^. Osteogenesis is reduced in femoral head necrotic zone as a result of decreased mesenchymal stem cells^19^. Thus, it is necessary to supplement stem cells or other osteogenesis factors. Patients with greater numbers of progenitor cells transplanted during core decompression procedures have a better prognosis reported by Hernigou *et al*.^41^. Bone marrow enrichment technology could substantially increase the number of BMMCs or osteogenesis factors available for transplantation. Recent research shows satisfactory results of novel actions of bisphosphonates in osteoblast preservation and osteocyte viability and pre‐treatment with bisphosphonate enhancing osteogenesis of bone marrow mesenchymal stem cells^22^, ^42^. Kang *et al*. demonstrated the efficacy with BP + core decompression^13^. A study from HSS hospital concluded that BP + core decompression + MSC are effective in delaying collapse within the first 24 months after diagnosis^32^.

In this study, we reported a relatively good mid‐term outcome with procedure of local administration of core decompression + BP + enriched BMMCs. The outcomes are comparable to that of the other complicated osteotomy surgeries^27^. Although 14 hips collapsed and had a deterioration, only six hips were converted to THA. The patients' choice to proceed with THA is multivariate and dependent on a careful discussion between the patient and their surgeon. For the young, they tend to postpone THA when presented with other minimally invasive alternatives or when the disease can be tolerable. Therefore, the indications for THA are not entirely objective and we use imaging progression as the main evaluation index. Our results show that the novel method may prevent or at least delay collapse progression. It may offer an alternative for the treatment of ONFH. In our study, the mean follow‐up time was more than 5 years (69.1 months). This is also the advantage of the results of this study.

### Limitation

There are several limitations of the present study. Our study was retrospective and thus maintains the biases inherent to such a study design. The sample size of this study is not large enough and there was no control group. Thus, future prospective randomized, controlled, multicenter studies are warranted to determine the efficacy of this treatment strategy in the long term. Last but not the least, the optimal dosage for local administration of bisphosphonate still needs to be further explored.

### Conclusion

Core decompression with local administration of zoledronate and enriched BMMCs could relieve the pain, delay the progression of collapse, and postpone the time of total hip arthroplasty in patients of early and mid‐term ONFH. This remedy might provide an alternative to preserve the affected hip.

## Compliance with Ethical Standards

This article was approved by the Medical Ethics Committee of Chinese PLA General Hospital.

## Supporting information


**Table S1.** Association Research Circulation Osseous (ARCO) Classification System 2019 revised version.
**Table S2.** Japanese Investigation Committee (JIC) classification system.
