# Supplementary material for: Core Decompression with Local Administration of Zoledronate and Enriched Bone Marrow Mononuclear Cells for Treatment of Non‐Traumatic Osteonecrosis of Femoral Head
Source: Orthop Surg. 2021 Oct 18;13(6):1843–52. doi: 10.1111/os.13100 (PMC8523758; doi:10.1111/os.13100)
Supplement: Supplementary file 1 — Table S1. Association Research Circulation Osseous (ARCO) Classification System 2019 revised version. Table S2. Japanese Investigation Committee (JIC) classification system. [file OS-13-1843-s001.docx]

**Supplementary**

Table S1. Association Research Circulation Osseous (ARCO) Classification System 2019 revised version

| **Stage** | **Findings** |
| --- | --- |
| I | X-ray is normal, but either magnetic resonance (MRI) or bone scan is positive |
| II | X-ray abnormal (subtle signs of osteosclerosis, focal osteoporosis, or cystic change in the femoral head), but without any evidence of subchondral fracture, fracture in the necrotic portion, or flattening of the femoral head |
| IIIa (early III) | fracture in the subchondral or necrotic zone as seen on x-ray or computed tomography (CT); early, femoral head depression ≤ 2 mm |
| IIIb (late III) | late, femoral head depression > 2 mm |
| IV | X-ray evidence of osteoarthritis with accompanying joint space narrowing, acetabular changes, and/or joint destruction. |

Table S2. Japanese Investigation Committee (JIC) classification system

| **Type** | **Description** |
| --- | --- |
| A | lesions occupied the medial one-third or less of the weight-bearing |
| B | lesions occupied medial two-thirds or less |
| C1 | lesions occupied more than the medial two-thirds but not extending laterally to the acetabular edge |
| C2 | lesions occupied more than the medial two-thirds and extending laterally to the acetabular edge |
